# Toward Replacing Late Gadolinium Enhancement With Artificial Intelligence Virtual Native Enhancement for Gadolinium-Free Cardiovascular Magnetic Resonance Tissue Characterization in Hypertrophic Cardiomyopathy

**DOI:** 10.1161/CIRCULATIONAHA.121.054432

**Published:** 2021-07-07

**Authors:** Qiang Zhang, Matthew K. Burrage, Elena Lukaschuk, Mayooran Shanmuganathan, Iulia A. Popescu, Chrysovalantou Nikolaidou, Rebecca Mills, Konrad Werys, Evan Hann, Ahmet Barutcu, Suleyman D. Polat, Hypertrophic Cardiomyopathy Registry (HCMR) Investigators, Michael Salerno, Michael Jerosch-Herold, Raymond Y. Kwong, Hugh C. Watkins, Christopher M. Kramer, Stefan Neubauer, Vanessa M. Ferreira, Stefan K. Piechnik

**Affiliations:** 1 Oxford Centre for Clinical Magnetic Resonance Research, Oxford Biomedical Research Centre National Institute for Health Research, Division of Cardiovascular (Q.Z., M.J.B., E.L., M.Shanmuganathan, I.A.P., C.N., R.M., K.W., E.H., A.B., S.D.P., H.C.W., S.N., V.M.F., S.K.P.); 2 Radcliffe Department of Medicine (Q.Z., M.J.B., E.L., M. Shanmuganathan, I.A.P., C.N., R.M., K.W., E.H., H.C.W., S.N., V.M.F., S.K.P.), University of Oxford, UK.; 3 Department of Medicine, University of Virginia Health System, Charlottesville, VA (M.Salerno, C.M.K.).; 4 Cardiovascular Division, Department of Medicine, Brigham and Women’s Hospital, Harvard Medical School, Boston, MA (M.J-H., R.Y.K.).

**Keywords:** artificial intelligence, cardiomyopathy, hypertrophic, contrast media, deep learning, gadolinium, magnetic resonance imaging

## Abstract

**Background:**

Late gadolinium enhancement (LGE) cardiovascular magnetic resonance (CMR) imaging is the gold standard for noninvasive myocardial tissue characterization but requires intravenous contrast agent administration. It is highly desired to develop a contrast agent–free technology to replace LGE for faster and cheaper CMR scans.

**Methods:**

A CMR virtual native enhancement (VNE) imaging technology was developed using artificial intelligence. The deep learning model for generating VNE uses multiple streams of convolutional neural networks to exploit and enhance the existing signals in native T1 maps (pixel-wise maps of tissue T1 relaxation times) and cine imaging of cardiac structure and function, presenting them as LGE-equivalent images. The VNE generator was trained using generative adversarial networks. This technology was first developed on CMR datasets from the multicenter Hypertrophic Cardiomyopathy Registry, using hypertrophic cardiomyopathy as an exemplar. The datasets were randomized into 2 independent groups for deep learning training and testing. The test data of VNE and LGE were scored and contoured by experienced human operators to assess image quality, visuospatial agreement, and myocardial lesion burden quantification. Image quality was compared using a nonparametric Wilcoxon test. Intra- and interobserver agreement was analyzed using intraclass correlation coefficients (ICC). Lesion quantification by VNE and LGE were compared using linear regression and ICC.

**Results:**

A total of 1348 hypertrophic cardiomyopathy patients provided 4093 triplets of matched T1 maps, cines, and LGE datasets. After randomization and data quality control, 2695 datasets were used for VNE method development and 345 were used for independent testing. VNE had significantly better image quality than LGE, as assessed by 4 operators (n=345 datasets; *P*<0.001 [Wilcoxon test]). VNE revealed lesions characteristic of hypertrophic cardiomyopathy in high visuospatial agreement with LGE. In 121 patients (n=326 datasets), VNE correlated with LGE in detecting and quantifying both hyperintensity myocardial lesions (*r*=0.77–0.79; ICC=0.77–0.87; *P*<0.001) and intermediate-intensity lesions (*r*=0.70–0.76; ICC=0.82–0.85; *P*<0.001). The native CMR images (cine plus T1 map) required for VNE can be acquired within 15 minutes and producing a VNE image takes less than 1 second.

**Conclusions:**

VNE is a new CMR technology that resembles conventional LGE but without the need for contrast administration. VNE achieved high agreement with LGE in the distribution and quantification of lesions, with significantly better image quality.

## Abstract 2

Supplemental Digital Content is available in the text.

**Clinical Perspective**

**What Is New?**

- Virtual native enhancement (VNE) is a new deep learning–driven cardiovascular magnetic resonance (CMR) technology that generates images closely resembling conventional late gadolinium enhancement without the need for gadolinium-based contrast agent; in other words, it serves as a “virtual contrast agent” and produces “virtual late gadolinium enhancement” images.
- VNE images achieve high agreement with late gadolinium enhancement in the visuospatial distribution and quantification of lesions, with significantly better image quality than late gadolinium enhancement.
- VNE offers a new CMR tissue characterization technology that can significantly reduce scan time and obviate the need for contrast agent.

**What Are the Clinical Implications?**

- For hypertrophic cardiomyopathy patients, VNE can obviate repeated administration of gadolinium-based contrast agent in serial CMR scans for monitoring disease progression.
- While currently validated in hypertrophic cardiomyopathy, there is a clear pathway to extend VNE to characterize a wider range of cardiac pathologies.
- The VNE technology has the potential to change the current paradigm for CMR imaging, as it may allow significantly faster, lower-cost, and contrast-free CMR scans, enabling frequent monitoring of myocardial tissue changes.

**Editorial, see p 600**

Late gadolinium enhancement (LGE) cardiovascular magnetic resonance (CMR) imaging is well validated for detecting focal myocardial lesions and fibrosis in a variety of cardiovascular diseases.^1–5^ The presence and extent of LGE is independently associated with adverse outcomes, including in hypertrophic cardiomyopathy (HCM).^6–11^ However, LGE requires intravenous injection of a gadolinium-based contrast agent (GBCA), which is cautioned in patients with severe kidney failure and contraindicated in those with known GBCA allergy.^12^ Eliminating the need for GBCA administration could significantly shorten scan times, reduce costs of associated consumables, shorten patient preparation time, and circumvent the need for physician presence.

Native (precontrast) CMR modalities are alternative means for tissue characterization without the need for GBCA. Cine imaging consists of a sequence of images at different cardiac phases to assess the cardiac structure and motion. Native T1 mapping estimates the T1 (proton spin-lattice) relaxation time of tissues on a pixel-by-pixel basis. Native T1 mapping exhibits sensitivity to a variety of cardiac diseases,^13^ including early myocardial changes in HCM.^14–16^ Abnormal T1 signals correlate to areas of LGE and on histopathology in models of focal and diffuse fibrosis.^17–19^ Native T1 mapping appears the most promising GBCA-free technique for revealing intrinsic imaging signals associated with myocardial abnormalities seen in LGE; however, the clinical utility of T1 mapping has been hindered largely by a lack of standardized interpretation and postprocessing, confounding factors, and diagnostic specificity.^13,20^

We hypothesized that native T1 maps may be transformed into visually diagnostic images similar to LGE images. In this work, using novel artificial intelligence (AI) approaches, a virtual native enhancement (VNE) imaging technology was developed, which exploits and enhances existing contrast and signals within the native T1 maps and cine frames, and displays them in a standardized presentation. The VNE imaging was then validated by comparison against matching LGE for image quality, visuospatial agreement, and myocardial lesion quantification. The approach was developed first in HCM, partly because it is an important indication for LGE assessment in its own right, but also because its features of regional heterogeneity and diverse tissue remodeling processes make it a good test case for a wide range of cardiac pathology.

## Methods

The anonymized test data that support the findings of this study are available from the corresponding author on reasonable request and subject to multicenter Hypertrophic Cardiomyopathy Registry (HCMR) committee approval.

### Deep Learning Method for Virtual Native Enhancement

**Figure 1.**
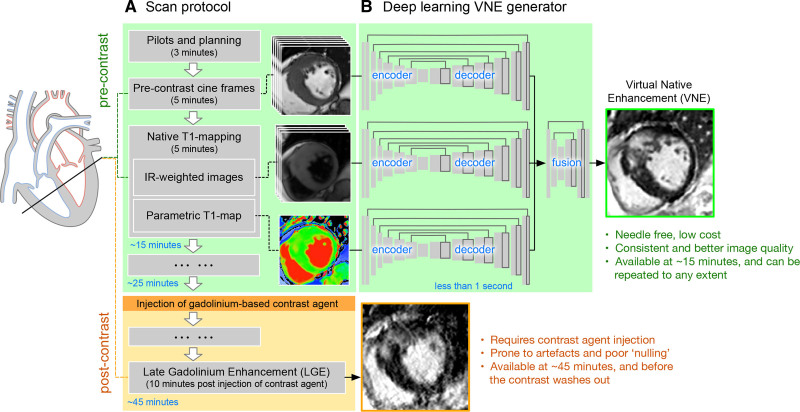
**Overview of the VNE imaging technology.A**, Simplified illustration of Hypertrophic Cardiomyopathy Registry scan protocol which includes native (precontrast) cine, T1 mapping (including native inversion recovery–weighted images), and conventional postcontrast late gadolinium enhancement. **B**, VNE generator. Native cardiovascular magnetic resonance images are input to 3 steams of encoder–decoder U-nets to extract feature maps, followed by a further neural network block to fuse all feature maps and derive a VNE image. Once trained, producing a VNE image takes <1 s. VNE indicates virtual native enhancement.

The proposed VNE technology uses 2 native components: native T1 mapping (including the native inversion recovery–weighted images) and precontrast cine frames of a cardiac cycle. Inversion recovery–weighted images and T1-maps provide image contrast and signal changes in myocardial tissue properties. Cine frames provide additional wall motion information and more defined myocardial borders. These images were input into a deep learning generator to derive a VNE image (Figure 1B).

#### Neural Network Design

The VNE generator has 3 parallel convolutional neural network streams to process cine frames, inversion recovery–weighted images and T1 maps, respectively. Each stream has an encoder–decoder U-net^21^ architecture (Figure 1B). The encoder successively computes image features from fine to coarse, providing a multiscale feature representation. The decoder combines the multiscale features to produce final feature maps. The 3 streams of feature maps by U-nets are concatenated and input into a further neural network block to fuse the information from multimodalities and produce a final VNE image.

#### Neural Network Training

The neural networks were trained using a modified conditional generative adversarial network approach,^22^ which optimizes the VNE generator together with a “discriminator.” This VNE application focused on the enhancement of native CMR signals and presented them as VNEs that resemble LGE images. This was achieved by defining the objective of the generator to produce VNE images that match LGE images in perceptual similarity—a higher deep learning feature comparison using a pretrained neural network (VGGNet)^23^—and are indistinguishable from LGE contrast images. The objective of the “discriminator” was to distinguish between VNE and LGE images. The 2 neural networks were trained in an adversarial manner. This strategy resulted in a trained generator that translated the existing native CMR signals into LGE representation. For reproducibility, full deep learning details are provided in the expanded Methods in the Data Supplement. Once trained, generating a VNE image takes ≈50 ms via a graphics processing unit, or 130 ms on a modern central processing unit. The generated VNE images have the same spatial resolution as the T1 maps.

### Materials for Validation of the Concept

**Figure 2.**
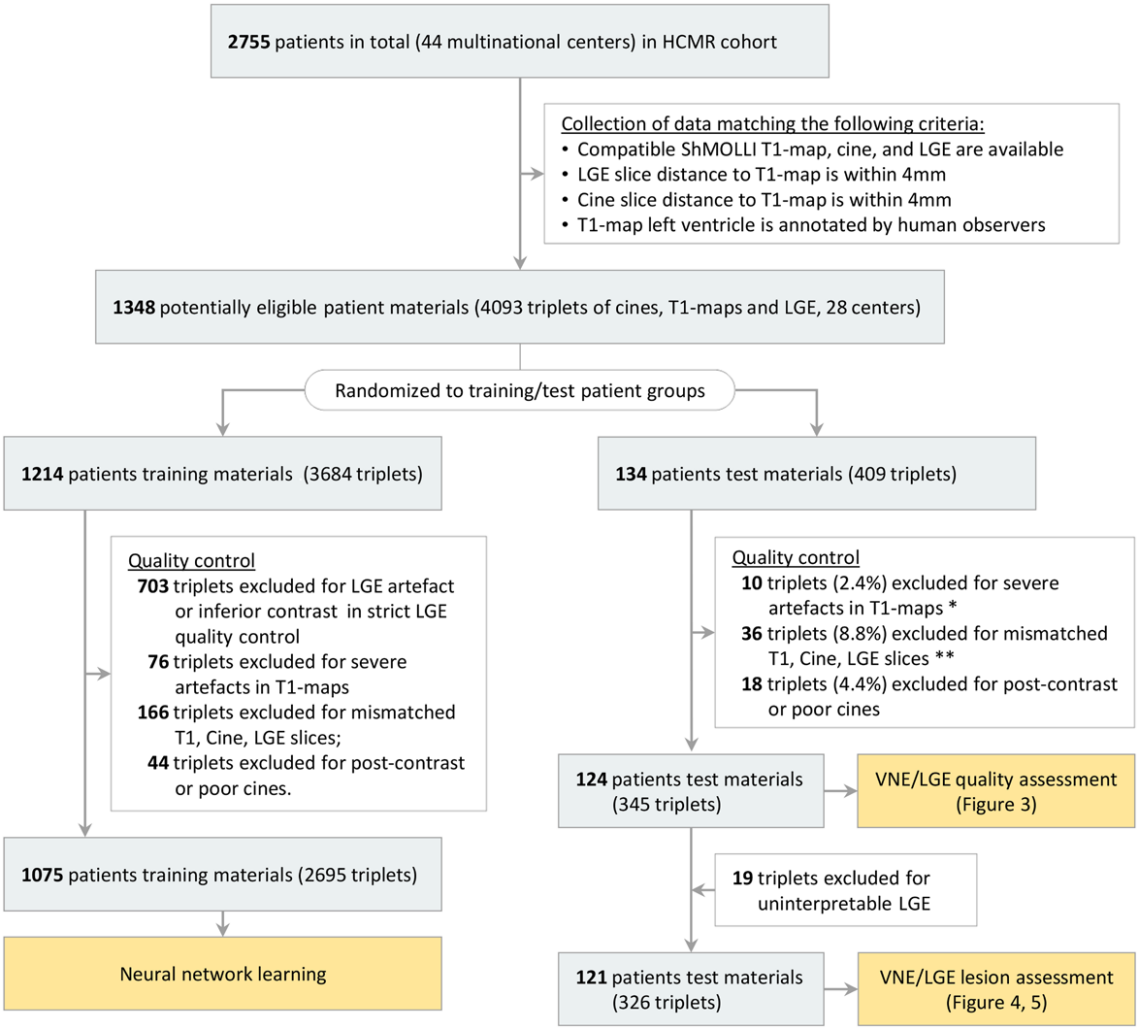
**Flow of patient material selection for developing and testing the virtual native enhancement technology.** *The excluded T1 maps (n=10) in testing materials are disclosed in Figure III in the Data Supplement. **Four of the 36 triplets were retrospectively excluded from analysis because of slice position mismatch and coil problems identified by consensus of 2 cardiovascular magnetic resonance experts (see Figure IV in the Data Supplement). These examples were not detected automatically using the predefined criteria in slice position matching, and were excluded after manual inspection. HCMR indicates Hypertrophic Cardiomyopathy Registry; LGE, late gadolinium enhancement; and VNE, virtual native enhancement.

CMR datasets from the large HCMR study^11^ were used. This study has institutional review committee approval and ethics approval, and all patients have given written consent. The HCMR^11^ scanning protocol included precontrast short-axis cine imaging (for assessing cardiac motion and structure) and native T1 mapping (quantitative pixel-wise maps of T1 relaxation time), followed by intravenous administration of 0.1 to 0.2 mmol/kg GBCA and LGE imaging at ≈10 minutes post-GBCA (Figure 1A). Each scan has typically 3 short-axis T1 maps and whole-heart short-axis coverage for cine and LGE. Short-axis native T1 maps (ShMOLLI [Shortened Modified Look-Locker Inversion recovery],^24^ a protocol checked using a phantom approach^25^), cines (before any administration of GBCA), and LGE images^26^ were collected. LGE images were acquired using a conventional and widely-available 2D breath-hold and segmented phase-sensitive inversion recovery method.^11^ The LGE phase-sensitive inversion recovery images were used for developing the deep learning models because of its consistent image appearance attributable to less sensitivity to the inversion time (TI) setting. T1 maps, cines, and LGE images were matched for slice orientation (slice plane cosine similarity >0.9) and position (slice location difference <4 mm) using an automated pipeline written in Python. Additional manual quality control was performed to exclude cases with severe artefacts and slice mismatch related to patient movement (Figure 2). All T1 maps have consistent pixel spacing (distance between pixel centers) of ≈1 mm in the datasets. Cine and LGE images were interpolated to match the pixel spacing, image position, and orientation to the T1 maps—therefore, a pixel-to-pixel match (see Figure I in the Data Supplement).

The CMR datasets were randomized into 2 independent groups for deep learning method development and testing (Figure 2). The development group was further divided into training (90%) and validation (10%) datasets. Deep learning models were blinded to the test group.

### Qualitative and Quantitative Evaluation of VNE and LGE

Three clinical assessors trained in CMR and 1 CMR radiographer scored the image quality of VNE and LGE guided by a 5-point categorical scale (“uninterpretable,” “poor quality,” “acceptable quality,” “good quality,” and “excellent quality”) that is intuitive for human operators (Figure II in the Data Supplement; interface by I.A.P.). Behind the interface, the score was recorded on a numeric scale between 0 to 100 for statistical analyses (see an example in Figure IIF of the Data Supplement). The quality aspects considered included motion artefacts, noise, image contrast, and clarity of tissue borders. The images were randomly shuffled and the operators were blinded to whether the image was VNE or LGE.

Semiautomated myocardial lesion quantification by VNE and LGE was performed as follows: epicardial and endocardial left ventricular contours were initialized automatically^27^ and adjusted manually on all images by an experienced operator (E.L., 10 years of experience in CMR image analysis) using MC-ROI (developed by S.K.P. in Interactive Data Language, version 6.1). A remote reference region of interest (ROI) without LGE was added and lesion burden was calculated using adaptations of the full width at half maximum (FWHM) method.^28^ Specifically, average signal intensities of remote myocardium ROI set the minimum values. Average signal intensities of the left ventricular blood pool center ROIs (avoiding papillary muscles) set the maximum values for consistency among cases with hyperintensity, intermediate-intensity signals, or no lesions. FWHM thresholding at the 50th percentile, despite superior reproducibility, has been reported to underestimate lesion burden in HCM.^29^ Therefore, progressive thresholds at the 25th and 12.5th percentiles were also used—referred to as full width at quarter maximum and full width at eighth maximum—respectively, to capture subtle, intermediate-intensity changes often seen in HCM.^30^ Lesion burden was quantified for each patient as the sum of lesion areas divided by the total left ventricular myocardial area in all available short-axis slices.

### Statistical Analysis

For image quality assessment, the test images were shuffled and scored blindly by all operators, with 20% random images scored repeatedly to calculate intraobserver variability (reported as SD) and intraclass correlation coefficients (ICC). The statistical significance of differences in VNE and LGE quality scores was analyzed using nonparametric Wilcoxon tests. Correlation between lesion burden quantification by VNE and LGE was assessed using linear regression coefficients and ICC. Bland–Altman analysis was performed to analyze any systematic differences between quantification by VNE and LGE. Statistical significance was defined as *P*<0.05.

## Results

### Study Population

Of the patient CMR datasets, 1348 met the selection criteria of having matched pre- and postcontrast images (Figure 2), providing 4093 triplets of T1 maps, cines, and LGE images from 28 multinational CMR sites. Quality control excluded postcontrast cines, T1 maps with severe artefacts, and mismatched slice locations in the triplets (Figure 2). After this, 2695 triplets of images (from 1075 patients) were available for training of the deep learning methods and 345 triplets (from 124 patients) for independent testing.

### Image Quality of VNE and LGE

**Figure 3.**
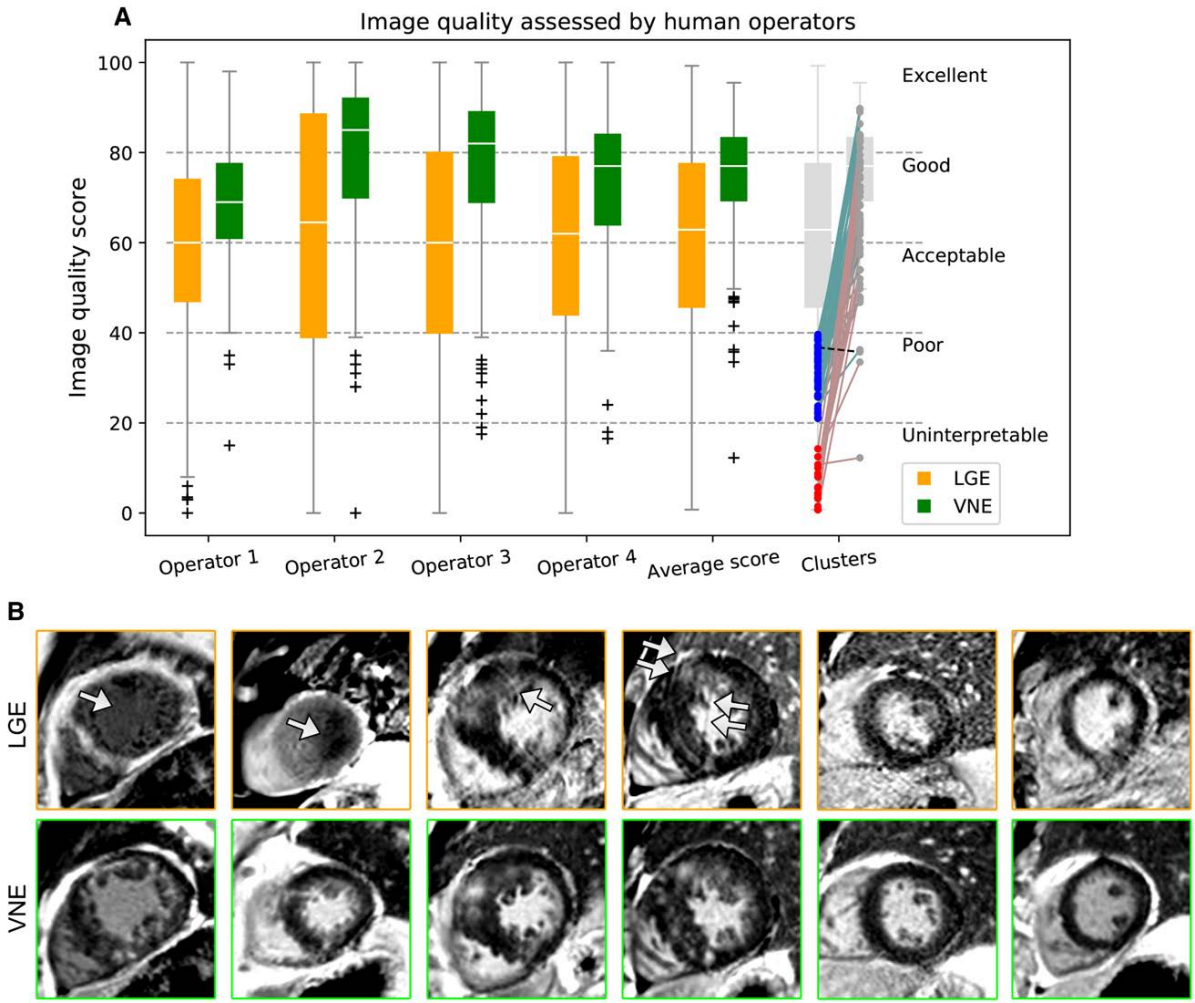
**VNE and LGE image quality assessment on 346 test materials (124 patients).A**, VNE provides significantly better image quality, as assessed by 4 blinded operators and their average scores (all *P*<0.001). For cases with “uninterpretable” (red clusters) or “poor” (blue) LGE images, VNE provides superior imaging quality in all but 1 case (dashed line). **B**, Examples of image quality improvement by VNE, which has more consistent appearance and defined borders. Arrows point to the LGE artefacts. LGE indicates late gadolinium enhancement; and VNE, virtual native enhancement.

On head-to-head comparisons, VNE provided significantly better image quality than LGE, as assessed by all 4 independent and blinded operators (*P*<0.001 [Wilcoxon test]) (Figure 3A). Intraobserver variability for the 4 operators was SD=5.9, 8.3, 6.9, and 7.3, and ICC=0.87, 0.88, 0.89, and 0.87. Interobserver variability was SD=8.5±0.7 and ICC=0.83±0.03. For “uninterpretable” (Figure 3A, red clusters; n=19) or “poor” (blue; n=53) LGE cases, VNE improved the quality of all but 1 image (Figure 3A, dashed line), and was scored as acceptable or better for all but 4 images. Conventional LGE can be affected by inaccurate TI selection and breathing artefacts attributable to patient fatigue at the last stage of long scanning sessions (Figure 3B, orange boxed). In contrast, VNE produced better, more consistent image quality (Figure 3B, green boxed). Additionally, VNE images have better-defined shapes and borders (Figure 3B, green boxed), likely inherited from cine images. Cases with “uninterpretable” LGE (5.5% [19 slices out of 345]) were excluded for lesion quantification assessment.

### Comparison of Myocardial Lesion Quantification Between VNE and LGE

**Figure 4.**
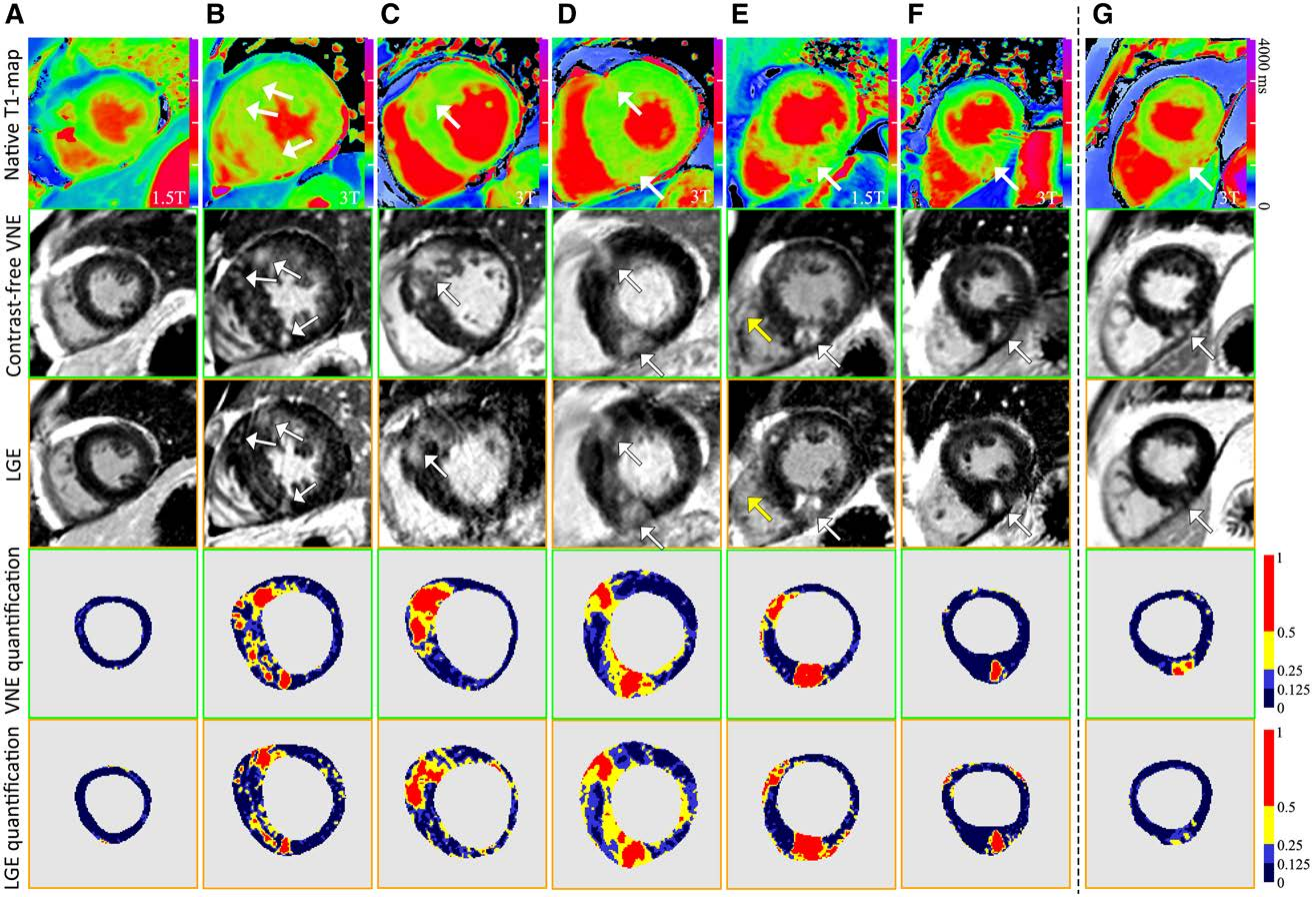
**Examples to illustrate visuospatial agreement between VNE and conventional LGE.** T1 colormaps (top row) were adjusted individually to highlight the T1 signals corresponding to VNE signals. The bottom 2 rows visualize lesion regions by VNE and LGE using progressive thresholding (full width at half, a quarter, and eighth maximum, ie, at 50th, 25th, and 12.5th percentiles) displayed with different colors. **A** through **F**, High visuospatial agreement was observed between VNE and LGE. Yellow arrows point to slightly different right ventricle sizes in VNE and LGE, suggesting patient movement between acquisitions. **G**, An example of VNE displaying subtle changes clearer than LGE. LGE indicates late gadolinium enhancement; and VNE, virtual native enhancement.

After exclusion of cases with “uninterpretable” LGE (Figure 3A, red clusters; n=19), lesion quantification was performed on 326 short-axis pairs of VNE and LGE images (from 121 patients). VNE lesions were in high visuospatial agreement with LGE, as visually assessed by 2 CMR experts independently (examples in Figure 4A–4F). The lesion regions defined by FWHM, full width at quarter maximum, and full width at eighth maximum methods (ie, thresholding at 50th, 25th, and 12.5th percentiles) were displayed with 3 progressive colors to visualize hyperintensity (red) and intermediate-intensity (yellow to light blue) abnormalities (Figure 4, bottom 2 rows). VNE revealed characteristic HCM lesions in hypertrophied segments and at the anterior and inferior right ventricular insertion points (Figure 4). Origins of these VNE signals can be seen in corresponding native T1-maps (Figure 4, top row). Despite matched slice position and orientation by image metadata between T1 maps and LGE, some T1 maps and their derived VNE have slightly different image appearance than the corresponding LGE (Figure 4E, yellow arrows). This may be because of slight positional differences attributable to patient movement between acquisitions of the T1 maps and LGE, despite meticulous checks for such effects (Figure 2; Figure IV in the Data Supplement).

**Figure 5.**
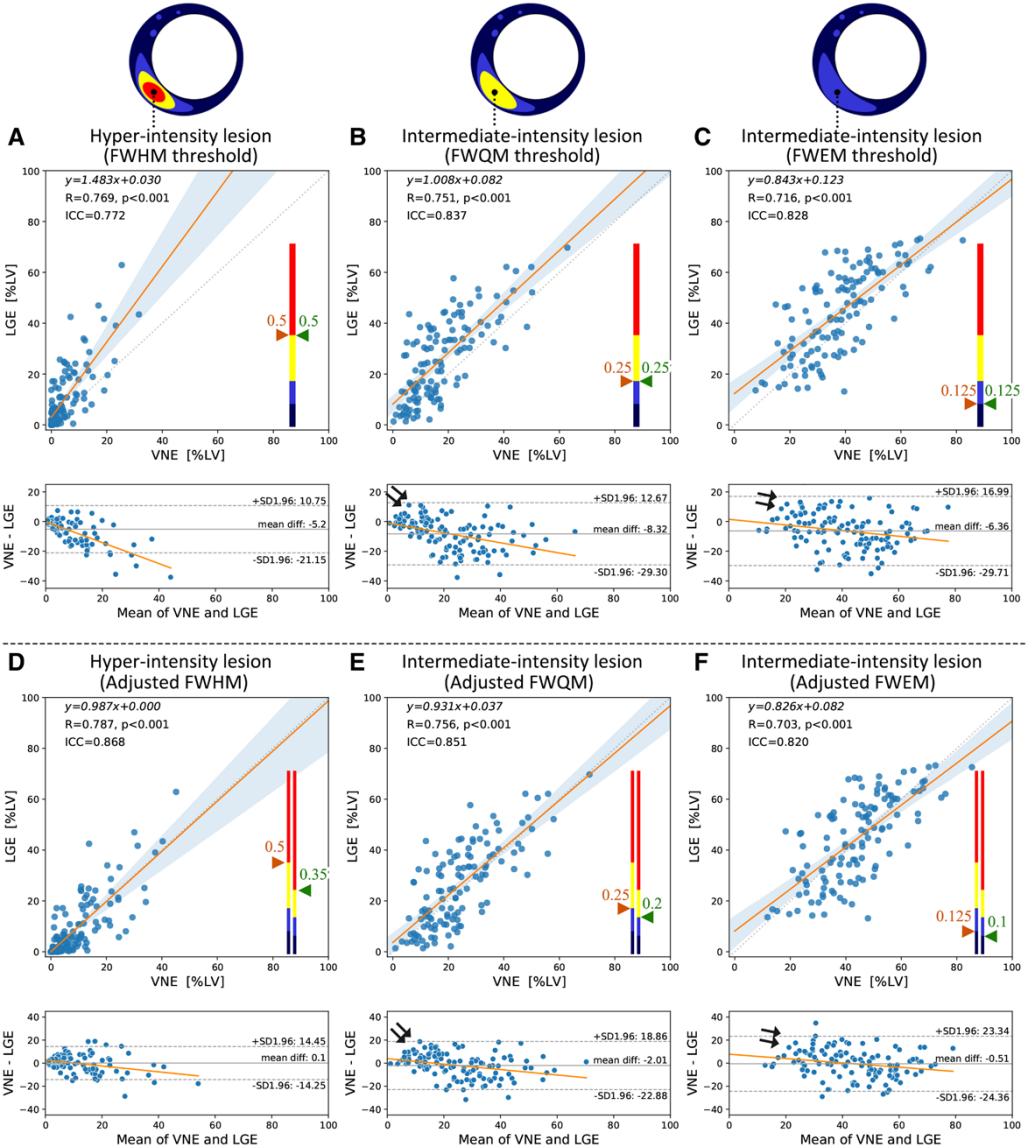
**VNE correlated strongly with conventional LGE in quantifying hyperintensity to intermediate-intensity lesions (left to right) in 121 test patients.A** through **C** use the same thresholding methods FWHM, FWQM, and FWEM (ie, thresholding at 50th, 25th, and 12.5th percentiles, reflecting hyperintensity to intermediate-intensity subtle lesions) for VNE and LGE. **D** through **F** use adjusted thresholding at 35th, 20th, and 10th percentiles for VNE. Threshold values are illustrated on color bars. Linear regression equations, correlation coefficient *R* values, and ICCs are provided. Bland–Altman plots demonstrate perceivable trends (arrowed) with associated clustering, suggesting enhanced signals in VNE for subtle lesions. FWEM, full width at eighth maximum; FWHM, full width at half maximum; FWQM, full width at quarter maximum; ICC, intraclass correlation coefficient; LGE, late gadolinium enhancement; LV, left ventricle; and VNE, virtual native enhancement.

In all 121 test patients, lesion burdens by VNE correlated strongly with LGE in both hyperintensity lesions (quantified by FWHM), and more subtle intermediate-intensity abnormalities (quantified by full width at quarter maximum and full width at eighth maximum). The linear correlation coefficient was *R*=0.77, 0.75, and 0.72, and ICC=0.77, 0.84, and 0.83 for FWHM, full width at quarter maximum, full width at eighth maximum, respectively (all *P*<0.001; Figure 5A–5C). Bland–Altman plots showed 5% to 8% average lower lesion burdens by VNE, with asymmetrical 95% CIs (upper bound, 11% to 17%; bottom bound, −21% to −30%) (Figure 5A–5C, bottom). These plots also revealed a perceivable skew, which may indicate enhanced signals in VNE for detecting subtle LGE lesions (Figure 5A–5C, arrowed), pending future validation. Figure 4G provides an example of subtle lesions in this range, in which VNE showed clearer lesion signals than the corresponding LGE, which also detected the lesions, albeit more subtly.

To achieve comparable lesion quantification between VNE and LGE, adjusted threshold values (eg, 35th, 20th, and 10th percentiles) can be used for VNE, to match with LGE using 50th, 25th, and 12.5th percentiles, respectively, when determining hyper- to intermediate-intensity abnormalities. The results (Figure 5D–5F) suggested that lesion quantification by VNE thresholding at the 35th percentile, for example, is directly comparable with LGE using the FWHM method (ie, 50th percentile), making this version of VNE highly promising to replace LGE in HCM lesion assessment. On the full test set, LGE and threshold-adjusted VNE reported similar average values of hyper- to intermediate-intensity lesions (LGE: 9.8%, 26.1%, 44.4% vs. VNE: 9.9%, 24.1%, 43.9%; Table I in the Data Supplement). VNE with adjusted thresholds also highlights the signals of subtle abnormalities for easier visualization (Figure 5E and 5F, arrowed). There were no false-positive VNE cases, where good quality VNE had introduced lesions in patients with good quality negative LGE by the conventional contrast–enhanced method.

## Discussion

In this work, an AI technology was presented that translates native T1 maps (together with cines) into the widely-recognized presentation of LGE, a format immediately ready for standard clinical interpretation. The AI deep learning is effectively acting as a “virtual contrast agent” that enhances the native CMR. In other words, it produces a “virtual LGE” image without the need for gadolinium. This work showed that: (1) VNE images had significantly better quality than LGE images; and (2) lesion burden quantification by VNE correlated well with LGE, both on a visuospatial (Figure 4) and quantitative (Figure 5) basis. The VNE technology has the potential to change the current paradigm for CMR imaging, as it may allow significantly faster, lower-cost and contrast-free CMR scans, enabling frequent monitoring of myocardial tissue changes.

### Advantages of VNE Over LGE

Conventional LGE is dependent on intravenous administration of GBCA and requires at least 10 minutes post-GBCA to develop the contrast redistribution.^31^ LGE image quality is dependent on appropriate adjustment of TI, although the phase-sensitive inversion recovery technique is less sensitive to TI setting. In comparison, VNE requires no intravenous access or GBCA, is derived from native imaging, and can be repeated as required to confirm findings and ensure sufficient image quality, without concerns about contrast agent wash-out. VNE uses readily available conventional cine and T1 mapping sequences, which can be completed within 15 minutes, limiting the likelihood of image artefacts attributable to patient fatigue. VNE showed significantly better image quality and more consistent image contrast than LGE (Figure 3).

### Lesion Assessment by VNE and LGE

VNE showed strong agreement with LGE in myocardial lesion visuospatial distribution and quantification. Intermethod comparison (ICC, 0.77–0.87; 95% CI, ≈20%) appears to be excellent in view of the reported LGE intramethod variability of ICC (0.88^29^) and interlaboratory inconsistencies of 10% to 15%.^32^ Above the linear correlation trend, there is a perceivable skew of enhanced signals in VNE (Figure 5, arrowed). While much work remains to confirm the clinical utility of detecting subtle lesions (often also seen in LGE), this sensitivity appears to arise directly from features of native T1 mapping, in line with literature reporting sensitivity of T1 to early myocardial changes in HCM patients.^14,15,33^

### Deep Learning Contrast Enhancement Mechanism

The concept of deep learning contrast enhancement emerged very recently with the advancement in AI methods. In CMR, although the term “synthetic LGE” (generated by deep learning) exists,^34–36^ it was designed for multimodal image registration and not the generation of lesion signals. In brain magnetic resonance imaging, “virtual gadolinium enhancement”^37,38^ aimed to reduce the contrast dose while predicting the full-dose image signals, but with notable degradation in quality and sensitivity, and with no application to image the heart.

CMR is inherently multimodal, with each modality demonstrating unique sensitivities to certain pathophysiologies. Previous T1 mapping development suggested that pursuing the exact reproduction of conventional modalities (such as LGE) may hinder the potential of the new technology to detect pathologies over standard methods.^39,40^ Therefore, VNE focused on enhancing existing native CMR signals. As a result, in addition to good agreement with LGE, the VNE technology also better displays the subtle lesions often seen in HCM patients.

### Limitations and Future Work

The current VNE is trained on LGE phase-sensitive inversion recovery images typically obtained >10 minutes post–GBCA administration.^11^ Separate training is required to predict other LGE pulse sequences and postcontrast images, for example, early gadolinium enhancement, first pass perfusion, and extracellular volume fraction mapping. In collaboration with magnetic resonance vendors, we plan to implement VNE as an inline sequence on the scanner to allow immediate VNE generation after cine and T1 map acquisitions. It can also be implemented for rapid offline analysis by third-party software vendors.

Individual imaging features may distinguish LGE from VNE and introduce observer bias in image quality assessment; reassuringly, there were no differences between operators who were aware of the study design (M.K.B., M. Shanmuganathan) and those who were not (C.N., R.M.). The VNE–LGE agreement appeared to be higher with better image quality (Figure V in the Data Supplement); future work is needed to test the association between image quality and diagnostic accuracy. The mechanism(s) of how each precontrast component contributes to the VNE signals is to be investigated using deep learning visualization techniques.^41,42^

Before recommendation for wide clinical use of VNE, further work is planned to link VNE-detected signals to patient outcomes. VNE may be expanded in future by adding more native modalities, such as T2 mapping or magnetic resonance fingerprinting.^43^ VNE variants developed on different cardiac disease datasets and native modalities can potentially differentiate between pathophysiologic changes, such as edema, fibrosis, and microvascular obstruction.

### Clinical Impact

#### T1 Mapping Interpretation

Clinical translation of T1 mapping is hindered by nonstandardized image interpretation methods.^44^ VNE has now addressed this challenge by translating T1 maps into the common language of LGE, whose interpretation is widely accepted and understood in routine clinical practice. Further, it provides a deep learning framework to enhance T1 maps with additional native CMR modalities, demonstrated in this work with precontrast cines.

#### HCM Assessment

The proposed VNE technology is of potentially high clinical impact to HCM patients, who often undergo serial CMRs to monitor disease progression. VNE can obviate repeated administration of GBCA and allow more frequent CMR follow-ups. Subject to further validation by the planned HCMR study outcomes, the apparent VNE signals to subtle lesions (inherited from T1 mapping) may potentially lead to better HCM risk stratification and treatment, especially given new, potentially disease-modifying therapies for HCM on the horizon.^45–48^

#### GBCA-Free CMR

By expanding the training material to a wider range of pathologies, it is envisioned that VNE could lead to a novel, GBCA-free CMR scanning protocol for myocardial tissue characterization, compatible with the standard clinical interpretation like for GBCA-based LGE. This could expand the capabilities of CMR to include patients in whom GBCAs are contraindicated, ultimately leading to increased patient benefit, satisfaction, and clinical throughput.

#### Cost Savings

Currently, the majority of CMR scans for tissue characterization requires intravenous access, the use of GBCA, related consumables, and patient preparation by trained staff. VNE is available immediately after native T1 mapping acquisition with no additional cost. Replacing LGE with VNE can significantly shorten the scan time to within 15 minutes, allowing twice as many patients to benefit from CMR at the same infrastructure capacity. The clinical impact and potential cost savings of popularizing this new CMR technology could be substantial.

### Conclusions

VNE imaging is a new CMR technology that resembles conventional LGE, without the need for GBCA administration. VNE achieved a high agreement with LGE in the visuospatial distribution and quantification of lesion burden, with significantly better image quality. While currently validated in the HCM population, there is a clear pathway to extend the technology to a wider range of myocardial pathologies. VNE has enormous potential to significantly improve clinical practice, reduce scan time and costs, and expand the reach of CMR in the near future.

## Sources of Funding

This article arises from research funded by the British Heart Foundation (project grant PG/15/71/31731), the National Heart, Lung, and Blood Institute (grant U01HL117006-01A1), the John Fell Oxford University Press Research Fund, and the Oxford British Heart Foundation Center of Research Excellence (grant RE/18/3/34214). The authors acknowledge British Heart Foundation Clinical Research Training Fellowship (FS/19/65/34692), the National Institute for Health Research Oxford Biomedical Research Center at the Oxford University Hospitals National Health Service Foundation Trust, and the National Institutes of Health.

## Disclosures

QZ, SKP, VMF, EH, IAP have authorship rights for pending patent WO2021/044153: “Enhancement of medical images” (patent cooperation treaty filed September 2020; intellectual property owned and managed by Oxford University Innovations). SKP has patent authorship rights for US patent US20120078084A1: “Systems and methods for shortened Look Locker inversion recovery (ShMOLLI) cardiac gated mapping of T1” (granted March 15, 2016; intellectual property is owned and managed by Oxford University Innovations; the license exclusively transferred to Siemens Healthcare). CK received research grants from and consults for MyoKardia (now Bristol Myers Squibb) and Cytokinetics. KW is an employee of Circle Cardiovascular Imaging since 2019.

## Supplemental Materials

Data Supplement Figure I–V

Data Supplement Tables I

Expanded Methods

HCMR Investigators

## Supplementary Material


