# Supplementary material for: Toward Replacing Late Gadolinium Enhancement With Artificial Intelligence Virtual Native Enhancement for Gadolinium-Free Cardiovascular Magnetic Resonance Tissue Characterization in Hypertrophic Cardiomyopathy
Source: Circulation. 2021 Jul 7;144(8):589–99. doi: 10.1161/CIRCULATIONAHA.121.054432 (PMC8378544; doi:10.1161/CIRCULATIONAHA.121.054432)
Supplement: Supplementary file 1 [file cir-144-589-s001.pdf]

SUPPLEMENTAL MATERIAL

Towards Replacing Late Gadolinium Enhancement With Artificial Intelligence Virtual Native Enhancement For Gadolinium-free Cardiovascular Magnetic Resonance Tissue Characterization in Hypertrophic Cardiomyopathy

Qiang Zhang, Matthew K. Burrage, Elena Lukaschuk, Mayooran Shanmuganathan, Iulia A. Popescu, Chrysovalantou Nikolaidou, Rebecca Mills, Konrad Werys, Evan Hann, Ahmet Barutcu, Suleyman D. Polat, HCMR investigators, Michael Salerno, Michael Jerosch-Herold, Raymond Y. Kwong, Hugh C. Watkins, Christopher M. Kramer, Stefan Neubauer, Vanessa M. Ferreira and Stefan K. Piechnik

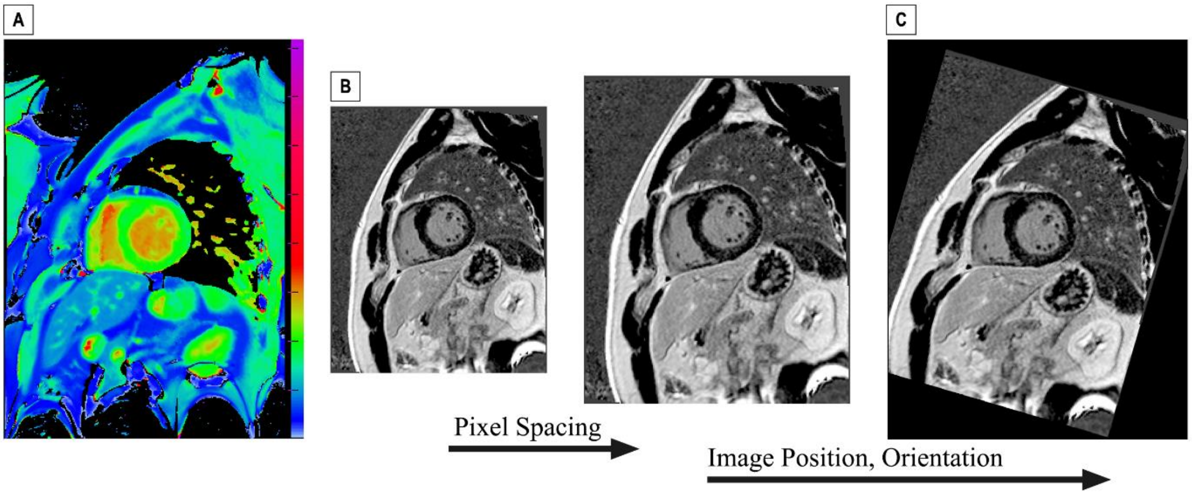

Figure I. Illustration of pre-processing LGE image to match with the T1-map pixel to pixel. In this example, the LGE image (B) (raw pixel spacing of 1.41mm) was interpolated to have matching pixel spacing (1.15mm), and transformed to have matching Image Position and Image Orientation with T1-map (A) based on the Dicom metadata. The resulting image (C) has the same size, pixel spacing, position and orientation with T1-map – therefore, a pixel-to-pixel match in the myocardium.

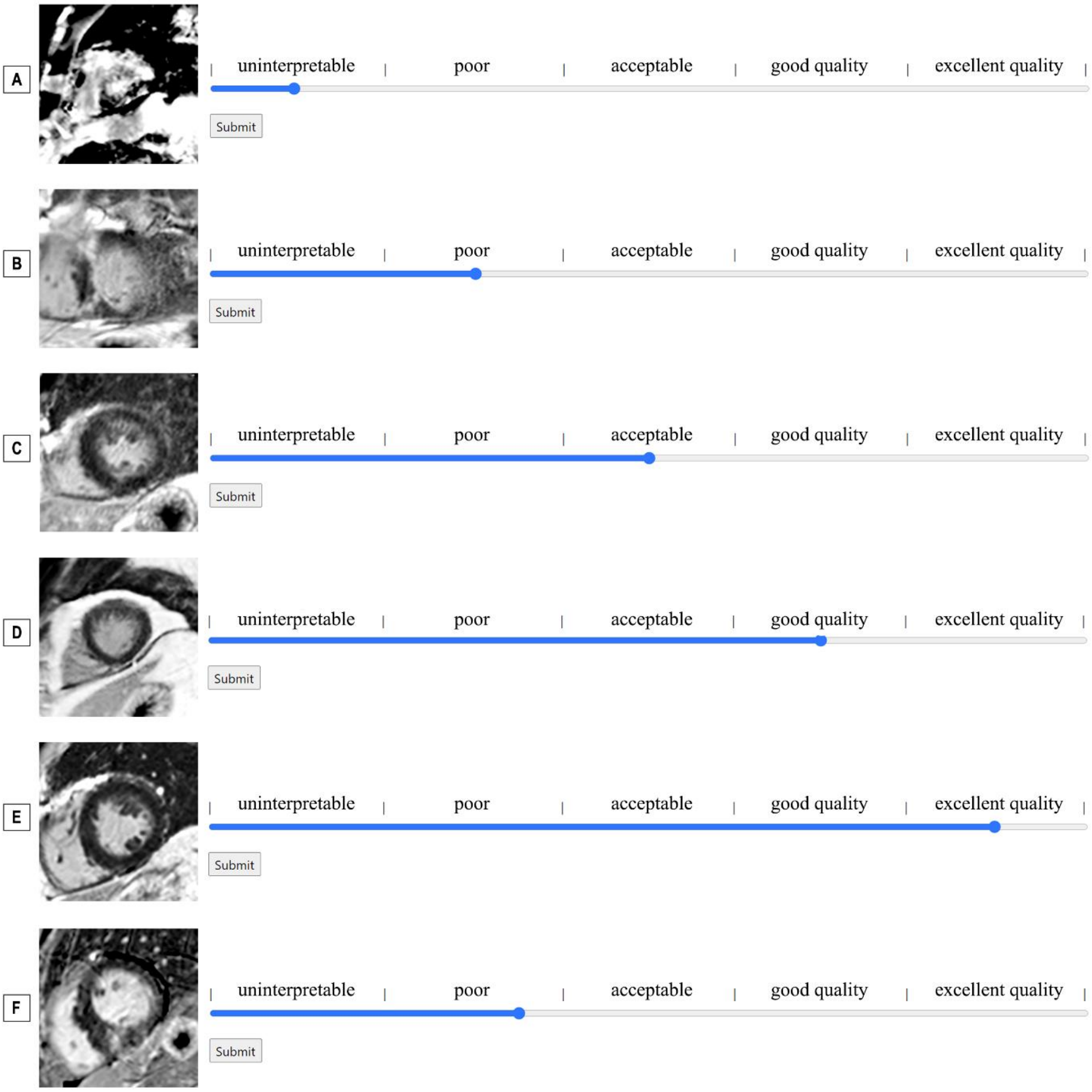

Figure II. LGE examples and their desired quality categories. Assessors were blinded to whether an image was VNE or LGE. (A-E) Five quality categories. (F) An example demonstrating the use of refined quality scoring. In the interface, human observers were allowed to register intermediate scores recorded on the scale of 0-100, e.g. '36' in this example. The motivation of this design was that categorical scales are typically more intuitive for human operators, whilst finer numerical scales are more suitable for statistical analyses.

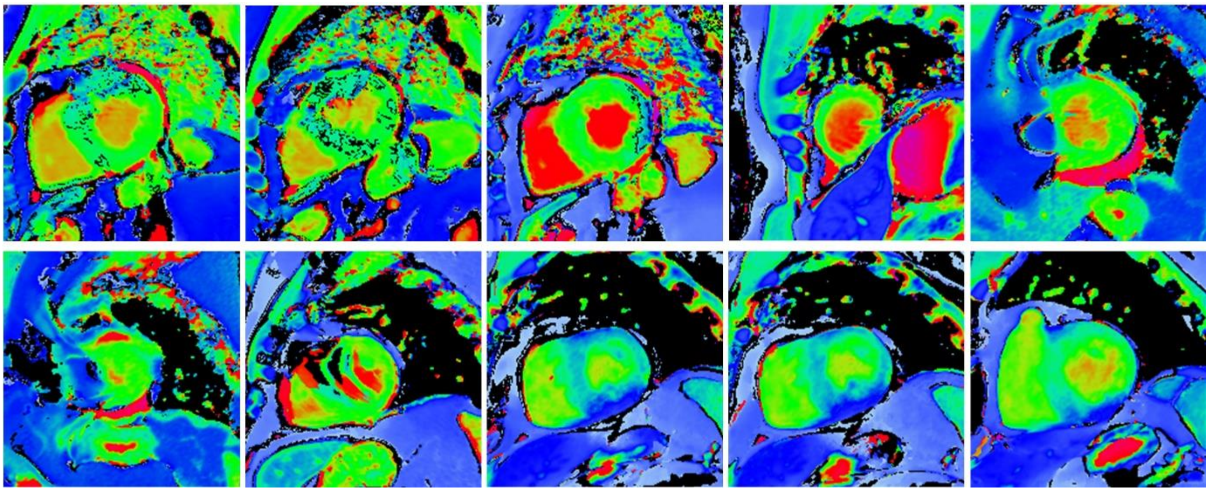

Figure III. T1-maps (n=10) in test materials that were excluded before VNE and LGE quality assessment. Severe artefacts are present in all these T1-maps, preventing any interpretation; therefore, these cases were manually excluded from subsequent analysis; see note 1 in Figure 2.

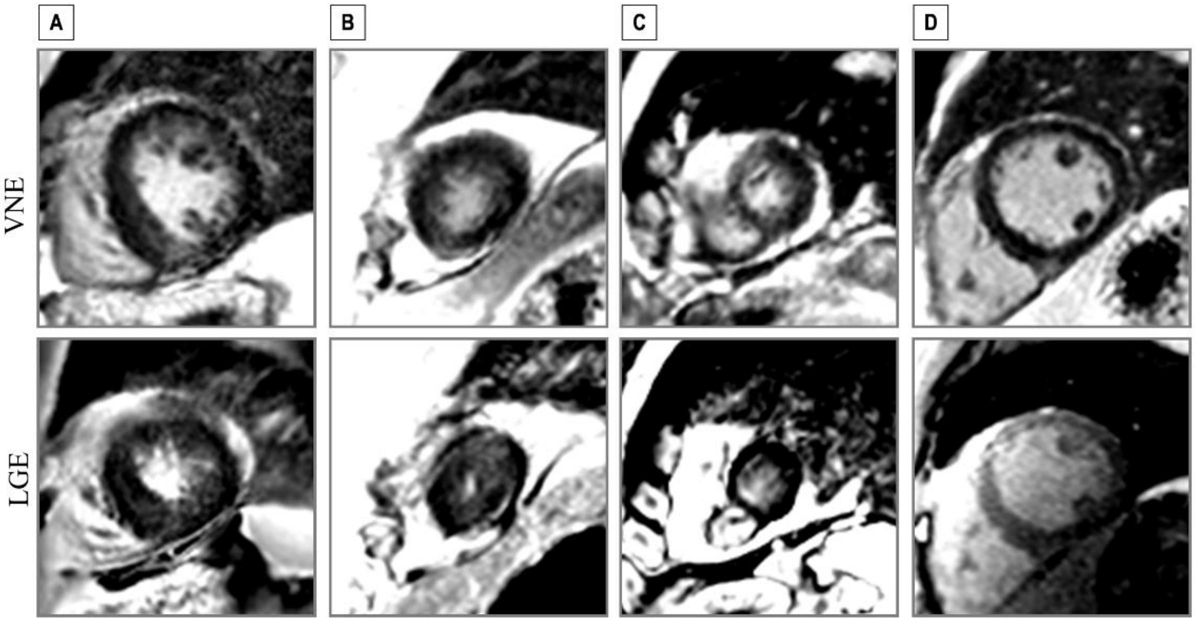

Figure IV. Image slices (n=4) in test materials that were retrospectively excluded in the analysis stage. These images were manually rejected by consensus of two experienced clinical operators comparing VNE and LGE, because of (A-C) large difference in slice locations between VNE and LGE, and (D) coil errors affecting the LGE image, as identified during the lesion assessment; see note 2 in Figure 2.

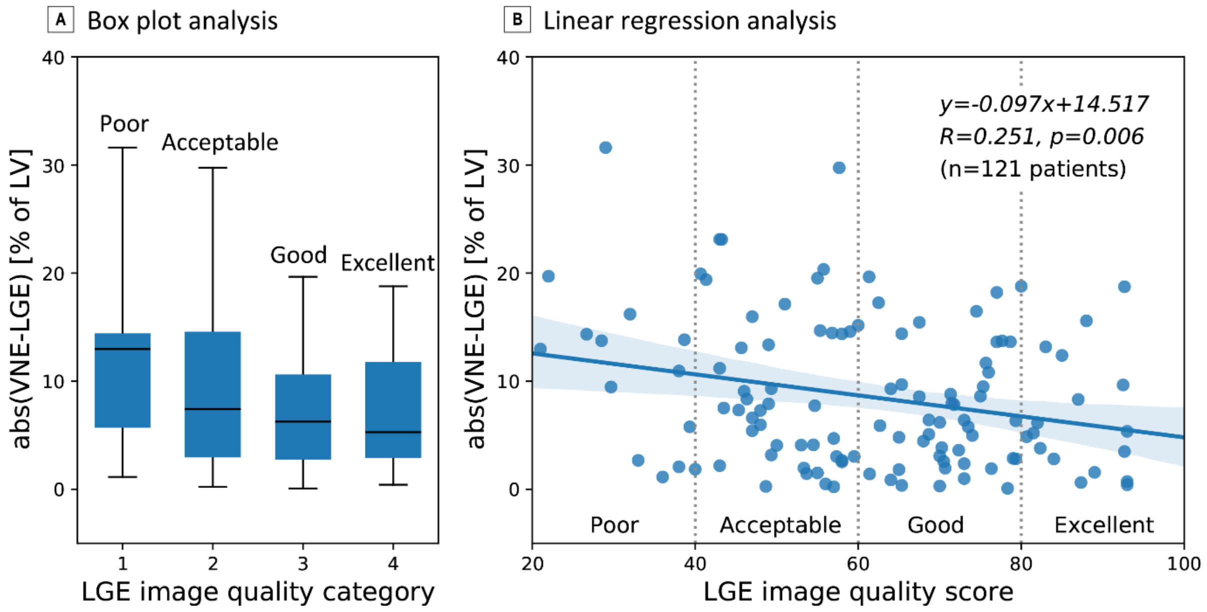

Figure V. Association between image quality and LGE-VNE agreement in quantifying lesion fractions, using threshold setting from Figure 5E as an example. The absolute difference between LGE and VNE quantification reduces with better image quality (p=0.006).

Table I. Lesion burden volume fractions by VNE and LGE in 121 test patients. Lesion regions are calculated using the same thresholding methods FWHM, FWQM, FWEM (i.e., thresholding at 50th, 25th, 12.5^{th} percentiles reflecting hyper-intensity lesions to intermediate-intensity subtle changes) for VNE and LGE, and also adjusted thresholding at 35th, 20th, 10th percentiles for VNE.

Modalities	LGE			VNE			Threshold-adjusted VNE		
Quantification methods (threshold values in percentile)	FWHM (50th)	FWQM (25th)	FWEM (12.5^{th})	FWHM (50th)	FWQM (25th)	FWEM (12.5^{th})	Threshold (35th)	Threshold (20th)	Threshold (10th)
Average lesion amount (% of LV)	9.8%	26.1%	44.4%	4.6%	17.7%	38.0%	9.9%	24.1%	43.9%
Lesion amount <5% of LV (n)	59	11	0	86	13	0	48	2	0
Lesion amount 5-10% of LV (n)	20	12	0	17	25	2	25	12	0
Lesion amount 10-15% of LV (n)	12	18	5	10	22	2	22	18	2
Lesion amount >15% of LV (n)	30	80	116	8	61	117	26	89	119

Supplemental Expanded Method

This supplemental material provides the deep learning method details for reproducibility of the technology. The virtual native enhancement (VNE) image generator was constructed based on multiple streams of U-nets [24], and trained it using the conditional generative adversarial network (cGAN) [25] strategy.

VNE generator

U-Net is a popular generative convolutional neural network (CNN) architecture that translates an input image into corresponding output masks or images. The VNE generator consists of three streams of 14-layer U-net blocks (**Figure VI**). Two streams utilize magnitude cine and inversion recovery images, with the third stream processing the T1-map. The final convolutional layer of these U-Nets is removed, such that they output feature maps from the second last layer. All feature maps are concatenated and passed onto a further 6-layer U-net block (**Figure VII**) to fuse the information and produce a VNE image.

T1-mapping (H×W×3)

Name	Kernel size	Filters	Stride	Input size	Output size	BN	Dropout
Conv2D_1	3x3	32	1	H×W×3	H×W×32	No	No
Conv2D_2	3x3	64	2	H×W×32	H/2×W/2×64	Yes	No
Conv2D_3	3x3	128	2	H/2×W/2×64	H/4×W/4×128	Yes	No
Conv2D_4	3x3	256	2	H/4×W/4×128	H/8×W/8×256	Yes	No
Conv2D_5	3x3	512	2	H/8×W/8×256	H/16×W/16×512	Yes	No
Conv2D_6	3x3	512	2	H/16×W/16×512	H/32×W/32×512	Yes	No
Conv2D_7	3x3	512	2	H/32×W/32×512	H/64×W/64×512	Yes	No
Upsample	-	-	-	H/64×W/64×512	H/32×W/32×512	-	-
Conv2D_8	3x3	512	1	H/32×W/32×512	H/32×W/32×512	Yes	Yes
Upsample	-	-	-	[H/32×W/32×512]×2	H/16×W/16×1024	-	-
Conv2D_9	3x3	512	1	H/16×W/16×1024	H/16×W/16×512	Yes	Yes
Upsample	-	-	-	[H/16×W/16×512]×2	H/8×W/8×1024	-	-
Conv2D_10	3x3	256	1	H/8×W/8×1024	H/8×W/8×256	Yes	Yes
Upsample	-	-	-	[H/8×W/8×256]×2	H/4×W/4×512	-	-
Conv2D_11	3x3	128	1	H/4×W/4×512	H/4×W/4×128	Yes	No
Upsample	-	-	-	[H/4×W/4×128]×2	H/2×W/2×256	-	-
Conv2D_12	3x3	64	1	H/2×W/2×256	H/2×W/2×64	Yes	No
Upsample	-	-	-	[H/2×W/2×64]×2	H×W×128	-	-
Conv2D_13	3x3	32	1	H×W×128	H×W×32	Yes	No

Concatenate → T1-mapping feature maps (H×W×32)

Figure VI. Configuration of the convolutional stream. The convolutional stream is used to extract feature maps from a native modality, in this example, T1-mapping. BN = batch normalization. Dropout rate was set as 0.2.

Cine, T1-map, T1W feature maps ([H×W×32]×3)

Name	Kernel size	Filters	Stride	Input size	Output size	BN	Dropout
Conv2D_1	3x3	32	1	[H×W×64]×3	H×W×32	No	No
Conv2D_2	3x3	64	2	H×W×32	H/2×W/2×64	Yes	No
Conv2D_3	3x3	128	2	H/2×W/2×64	H/4×W/4×128	Yes	No
Upsample	-	-	-	H/4×W/4×128	H/2×W/2×128	-	-
Conv2D_4	3x3	64	1	H/2×W/2×128	H/2×W/2×64	Yes	No
Upsample	-	-	-	[H/2×W/2×64]×2	H×W×128	-	-
Conv2D_5	3x3	32	1	H×W×128	H×W×32	Yes	No
Conv2D_6	3x3	1	1	[H×W×32]×2	H×W×1	No	No

VNE

Figure VII. Configuration of the CNN fusion block. The fusion block combines feature maps of multi-modalities and derive VNE images.

VNE generator training using cGAN

CGAN consists of two “adversarial” models: a generative model *G* and a discriminative model *D*. In this application, *G* is the VNE generator that produces the VNE images which resembles LGE, and *D* is a classification neural network (**Figure II**) that distinguishes between VNE and LGE images. *G* and *D* are trained simultaneously.

Objective

G and *D* are trained by optimizing the value of an objective function (**Figure**). Suppose there is a native CMR input x which is processed by *G* to produce the VNE image $G(x)$ that resemble the LGE image y. In this application, the objective for cGAN optimization can be expressed as an adversarial minimax game:

$$\min_G \max_D \left(\lambda_1 \|y - G(x)\|_1 + \lambda_2 \|VGG(y) - VGG\big(G(x)\big)\|_1 + \log\Big(1 - D\big(G(x)\big)\Big) + \log D(y)\right),$$

where *G* is optimized to minimize the objective function, while *D* is optimized to maximize the objective function. The first term is an *L1* loss that encourages the generator *G* to produce $G(x)$ that matches y pixel by pixel. Rather than exact replication of real LGE signal intensities, this VNE application focuses on enhancing the native CMR signals and translating the native images into the presentation of LGE. To account for this the second term is a perceptual loss [49] which calculates differences between high-level image feature representations of $G(x)$ and y. The features, denoted by $VGG\big(G(x)\big)$ and $VGG(y)$, are generated from the last convolutional layer of a 16-layer VGG network pre-trained on ImageNet [50]. In the third and fourth terms, $G(x)$ and y are input to the discriminator *D* which produces the “realness” labels

$D(y)$ and $D(G(x))$ as 1: “real” LGE or 0: “virtual” LGE. The objective of training D is to distinguish between real and virtual LGE images, i.e., to maximize the last two terms. Simultaneously, *G* is encouraged to produce VNE that cannot be distinguished from real LGE appearance by the discriminator *D*, i.e., to minimize the third term. The weighting parameters λ_1 and λ_2 are used to balance the magnitude of terms. In this application, a much lower $\lambda_1 = 20$ and higher $\lambda_2 = 200$ were set in order to enforce matching in perceptual features rather than pixel values. The strategy results in a trained generator that translates the existing native CMR signals into LGE image appearance.

To account for the inevitable position differences between native modalities and LGE used in training, additional modification was added to the first L1 loss term, to shift the LGE image locally and search for the best match:

$$\min_{i,j} \left\| y_{i,j} - G(x) \right\|_1,$$

where $i, j \in \{-10, -9 \ \ldots, 10\}$ denote the shift in pixels horizontally and vertically.

Optimization

To improve the robustness of the model, on-the-fly augmentation was employed on the training dataset, introducing uniformly distributed random rotation within ±5 degrees and translation within ±2 pixels around the manually annotated center of the LV cavity. The specifications of training CNN were: input size=128x128, batch size=1, learning rate=0.0002; Adam [51] was used as the optimizer. The networks were implemented in TensorFlow [52] and trained using an NVIDIA TITAN XP GPU, for 400 epochs, taking approximately 25 hours. Once trained, generating the test VNE images (n=349) takes 16.7 seconds on the GPU (0.05 seconds per image), or 43.5 seconds (0.13 seconds per image) on a 3.80GHz Intel Core i5 CPU.

VNE or LGE (H×W×1)

Name	Kernel size	Filters	Stride	Input size	Output size	BN	Activation
Conv2D_1	3x3	64	1	H×W×1	H×W×64	No	LeakyReLU
Conv2D_2	3x3	64	2	H×W×64	H/2×W/2×64	No	LeakyReLU
Conv2D_3	3x3	128	1	H/2×W/2×64	H/2×W/2×128	No	LeakyReLU
Conv2D_4	3x3	128	2	H/2×W/2×128	H/4×W/4×128	No	LeakyReLU
Conv2D_5	3x3	256	1	H/4×W/4×128	H/4×W/4×256	No	LeakyReLU
Conv2D_6	3x3	256	2	H/4×W/4×256	H/8×W/8×256	No	LeakyReLU
Dense	-	-	-	H/8×W/8×256	1024	No	LeakyReLU
Dense	-	-	-	1024	1	No	Linear

“Realness” score

Figure VIII. Configuration of the discriminator. The neural network discriminator is used in the conditional Generative Adversarial Network training approach.

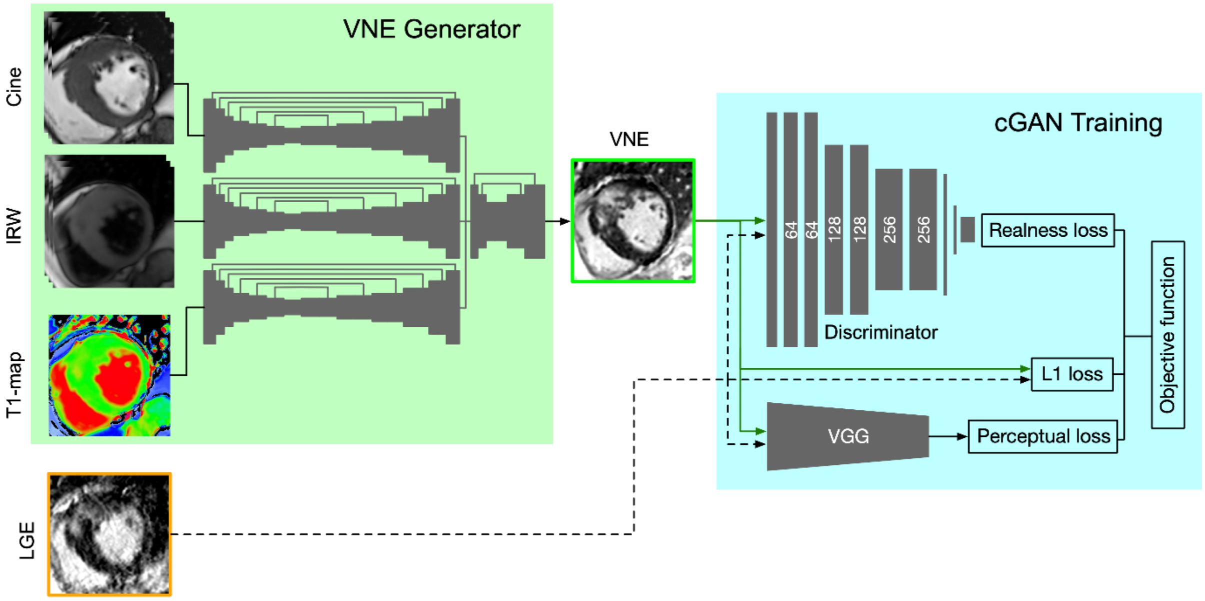

Figure IX. VNE neural network training using a conditional Generative Adversarial Network (cGAN) approach. IRW = inversion recovery-weighted images. VGG = VGG Neural Network.

Supplemental Content: HCMR investigators

(In alphabetical order)

Theodore Abraham, MD, Hypertrophic Cardiomyopathy Center of Excellence, Johns Hopkins University, Baltimore, MD;

Lisa Anderson, MD, St George's University Hospitals NHS Trust, London, United Kingdom;

Florian Andre, MD, Department of Medicine, Heidelberg University, Heidelberg, Germany;

Evan Appelbaum, MD, Departments of Medicine, Cardiovascular Division & Radiology, Beth Israel Deaconess Medical Center, Harvard Medical School, Boston, MA;

Camillo Autore, MD, Division of Cardiology, Department of Clinical & Molecular Medicine, St. Andrea Hospital, Sapienza University, Rome, Italy;

Colin Berry, MD, British Heart Foundation Glasgow Cardiovascular Research Center, Institute of Cardiovascular & Medical Sciences, University of Glasgow, UK;

Elena Biagini, MD, Cardio-Thoraco-Vascular Department, University Hospital of Bologna, Policlinico S. Orsola-Malpighi, Bologna, Italy;

William Bradlow, MD, Department of Cardiology, New Queen Elizabeth Hospital Birmingham, UK;

Chiara Bucciarelli-Ducci, MD, Bristol Heart Institute, Bristol National Institute of Health Research (NIHR) Biomedical Research Center, University Hospitals Bristol NHS Trust & University of Bristol, UK;

Amedeo Chiribiri, MD, PhD, Cardiovascular Division, Kings College London British Heart Foundation Center of Excellence, The Rayne Institute, St. Thomas Hospital Campus, London, UK;

Lubna Choudhury, MD, Division of Cardiology, Department of Medicine, Bluhm Cardiovascular Institute, Northwestern University Feinberg School of Medicine, Chicago, IL;

Andrew Crean, MD, Division of Cardiology, Peter Munk Cardiac Center, University Health Network, University of Toronto, Ontario, Canada;

Dana Dawson, MD, Aberdeen Cardiovascular & Diabetes Center, University of Aberdeen, UK;

Milind Y. Desai, MD, Department of Cardiovascular Medicine, Center for Radiation Heart Disease, Heart & Vascular Institute, Cleveland Clinic, Cleveland, OH;

Patrice Desvigne-Nickens MD, National Heart, Lung, and Blood Institute;

John DiMarco MD, PhD, University of Virginia Health System;

Eleanor Elstein, MD, Division of Cardiology, Department of Medicine, Royal Victoria Hospital, McGill University Health Center, Montreal, Quebec, Canada;

Andrew Flett, MD, Department of Cardiology, University Hospital Southampton NHS Foundation Trust, Southampton, UK;

Nancy Geller PhD, National Heart, Lung, and Blood Institute;

Stephen Heitner, MD, Oregon Health & Sciences University (OHSU), Division of Cardiovascular Medicine, Knight Cardiovascular Institute, Portland, OR;

Adam Helms, MD, Department of Internal Medicine, University of Michigan, Ann Arbor, MI;

Carolyn Ho, MD, Cardiovascular Division, Brigham and Womens Hospital, Boston, MA;

Daniel L. Jacoby, MD, Section of Cardiovascular Medicine, Department of Internal Medicine, Yale School of Medicine, New Haven, CT;

Han Kim, MD, Duke Cardiovascular Magnetic Resonance Center & Division of Cardiology, Duke University Medical Center, Durham, NC;

Bette Kim, MD, Mount Sinai West, Icahn School of Medicine at Mount Sinai, New York City, NY;

Dong-Yun Kim PhD, National Heart, Lung, and Blood Institute;

Eric Larose, MD, Quebec Heart & Lung Institute, Laval University, Quebec, Canada;

Masliza Mahmod, MD, Division of Cardiovascular Medicine, Radcliffe Department of Medicine, University of Oxford, UK;

Heiko Mahrholdt, MD, Department of Cardiology, Robert-Bosch-Krankenhaus, Stuttgart, Germany;

Martin Maron, MD, Hypertrophic Cardiomyopathy Center & Research Institute, Tufts Medical Center, Boston, MA;

Gerry McCann, MD, Department of Cardiovascular Sciences, University of Leicester, UK;

Michelle Michaels, MD, Erasmus University, Rotterdam, the Netherlands;

Saidi Mohiddin, MD, Barts Heart Center, The Cardiovascular Magnetic Resonance Imaging Unit, St Bartholomew’s Hospital, London, UK;

Sherif Nagueh, MD, Methodist DeBakey Heart & Vascular Center, Houston, TX;

David Newby, MD, Center for Cardiovascular Science, University of Edinburgh, UK;

Iacopo Olivotto, MD, Cardiomyopathy Unit & Genetic Unit, Careggi University Hospital, Florence, Italy;

Anjali Owens, MD, Center for Inherited Cardiovascular Disease, Division of Cardiovascular Medicine, Perelman School of Medicine, University of Pennsylvania, Philadelphia, PA;

F. Pierre-Mongeon, MD, Montréal Heart Institute, Canada;

Sanjay Prasad, MD, National Heart & Lung Institute, Imperial College London & Royal Brompton Hospital, London, UK;

Ornella Rimoldi, MD, Vita Salute University & San Raffaele Hospital, Milan, Italy;

Jeanette Schulz-Menger, MD, Charité, Medical Faculty of the Humboldt University, Experimental & Clinical Research Center and Helios Clinics, Cardiology, Berlin, Germany;

Mark Sherrid, MD, Hypertrophic Cardiomyopathy Program, Leon Charney Division of Cardiology, Department of Medicine, New York University School of Medicine, New York, NY;

Sven Plein, MD, PhD, Department of Biomedical Imaging Science, Leeds Institute of Cardiovascular and Metabolic Medicine, University of Leeds, UK;

Albert van Rossum, MD, Department of Cardiology, Amsterdam UMC, HZ Amsterdam, the Netherlands;

Jonathan Weinsaft, MD, Departments of Medicine & Radiology, Weill Cornell Medical College, New York, NY;

James White, MD, Calgary Foothills Medical Center, University of Calgary, Alberta, Canada;

Eric Williamson, MD, Department of Radiology, Mayo Clinic, Rochester, MN.
